# Medical and Physiological Commentaries

**Published:** 1841-04

**Authors:** 


### REVIEWS.

---

ART. III.—*Medical and Physiological Commentaries.* By MARTYN PAINE, M. D. A. M.
Vitæ tam vires, quam actiones expono.—*Biblia Naturæ.* Morborum quoque te causas et signa doceo.—*Virgil.* In two volumes. New York; Collins, Keese & Co., 254 Pearl street. London; John Churchill. 1840. pp. 1530, 8 vo.

THE work, whose title is announced in the above heading, is one of the most remarkable contributions that has been made to the medical literature of our country.

As an American production, not only is it unusual in its size, and in the abundance of its matter, it is still more unusuál on account of the extent and elaborateness of research, especially in books, and of the great amount and diversity of learning, both ancient and modern, by which it is characterized—and, as respects the *latter* quality, we might safely add *enriched.* Indeed in both qualities, if we mistake not, it may be pronounced *unique* in the medical annals of the United States. And in standing sufficiently correspondent to these are the magnitude, and bold ambitiousness of its aim and object—namely, TO EXPOUND, VINDICATE, AND DETERMINE, SOME OF THE FUNDAMENTAL PRINCIPLES, AND MOST IMPORTANT DOCTRINES, IN THE PHILOSOPHY OF MEDICINE.

How far our author has succeeded, in the accomplishment of this arduous and intrepid enterprise of mind, it might be hazardous, and not perhaps altogether becoming in us, to undertake to decide. An attempt of the kind might be thought to betray in us an unauthorized assumption, as respects our own competency to adjudicate and settle some of the highest and most intricate points of litigation in the science of medicine—perhaps we might say, *in any of the sciences.* For, of all the branches which compose the aggregate of human knowledge, that involving the principles and laws, which preside over living organized matter, and regulate its functions, especially when considered in its connexion with mind, is at once the most lofty and subtle, the most recondite and difficult of attainment.

In the movements of the universe of dead matter, whether contemplated in the magnitude of worlds, or the minuteness of atoms, there prevail an uniformity and certainty of action and change, which admit of calculation, and furnish a degree of fore-knowledge equally certain. Hence the branches of science embracing the phenomena and causes of these movements and their consequences, have received, by way of pre-eminence, the title of "*exact.*"

But in the functions of all forms of living matter, whatever may be its rank in the scale of being, not only is the case *otherwise;* it may almost be said to be directly the *reverse. There,* so different from each other, and, at the same time, so mutable and uncertain, are the kinds and degrees of action and its effects, that they *too frequently,* not to say *generally,* set calculation, and foresight, and confidence at defiance. Hence the impossibility of arriving, as relates to them, at definitive conclusions.

We speak not here of the *positive* condition and character of the principles and laws that govern the world of living matter. Those great sources and regulators of action and change are, *in themselves and in their relations to one another*, as certain, harmonious, and stable, as are any of the other elements or attributes of creation. And creation, being the work of an ALL-PERFECT BEING, is free itself from imperfection and fault. The irregularity and unstableness apparent in it, are *only* apparent. They exist only in relation to ourselves, on account of the insufficiency of our knowledge and comprehension of them. Should the period ever arrive (and it would be difficult to give a reason, why we should despair of its arrival,) when the natural causes which preside over the functions of living matter shall be as thoroughly understood, as are now the principles and laws of mathematics and mechanics, computations respecting their operations and the results of them, will be equally unerring.

For reasons such as these, we say, it might, not improperly perhaps, be deemed too assuming in us to attempt a positive decision of the correctness or incorrectness of the opinions and doctrines for which our author contends. And still more presuming and inadmissible might it be thought in us, were we to endeavor to fasten authoritatively our own decisions respecting those subjects on the minds of our readers, who have an equal right, and perhaps more ability, to form correct conclusions, than ourselves. Those members of the profession, who are desirous of attaining a thorough and competent knowledge of the work we are examining must peruse it themselves; and not merely "peruse" it, but make it a subject of laborious and accurate study. In no other way can their desire be gratified.

These considerations however furnish no good reason why we should decline submitting to our readers such opinions and thoughts on the general character of the whole production, as well as on the special character of some of its subdivisions, as a careful inspection of it has enabled us to form. Nor is it our intention to decline what we regard as a duty. On the contrary, we shall avail ourselves of the liberty and right conceded to reviewers, by the canons of criticism, to scrutinize strictly, not to say severely, several of the leading tenets of our author. And, in thus proceeding, though solicitous neither to injure nor offend, we shall notwithstanding, irrespectively of aught save truth and usefulness, speak of his writings, in matter and manner, as we may think they deserve. We shall adopt, as our motto, that which should be held sacred alike by the critic and the judge, *fiat justitia, ruat cœlum*, And if we obey not its mandate, our failure shall be the issue of an error in judgment.

The "Commentaries" of Dr. Paine have nothing systematic in their arrangement or form. They do not even constitute a continuous work. Nor could they be made available for such a work, except in the character of elementary ingredients. Yet are they intended, as already suggested, to have somewhat of a systematic effect, by illustrating, carrying out, and confirming certain leading and important doctrines in the philosophy of medicine. They consist of several very extensive and ponderous articles, or rather essays, and a few shorter and lighter ones, treating of subjects which have no immediate connexion with each other; and each essay is divided into "Sections," under which, subordinate points embraced in the scope of the leading disquisitions, are considered in detail. The subjects or titles of the essays are as follows:

*Vol. I.—Essay I.*

"VITAL POWERS.

II.

"PHILOSOPHY OF THE OPERATION OF THE LOSS OF BLOOD.

III.

"THE HUMORAL PATHOLOGY."

*Vol. II.—Essay I.*

"PHILOSOPHY OF ANIMAL HEAT.

II.

"PHILOSOPHY OF DIGESTION.

III.

"THEORIES OF INFLAMMATION.

IV.

"PHILOSOPHY OF VENOUS CONGESTION.

V.

"COMPARATIVE MERITS OF THE HIPPOCRATIC AND ANATOMICAL SCHOOLS.

VI.

"ON THE PRINCIPAL WRITINGS OF P. CH. A. LOUIS, M. D."

From these essays, pursuing the order of the preceding enumeration of them, it is our purpose, in our review of the "Commentaries," to make such extracts as may be deemed best calculated to communicate to our readers some knowledge of the general contents of the work, accompanied by remarks expressive of our own views of the subjects discussed, and of the principles and doctrines maintained in relation to them. With this design we shall begin with the essay on the

"VITAL POWERS."

Under this head Dr. Paine, like a warrior, confident alike of the rectitude of his cause and of his own powers to maintain it, promptly "shows his colors," and heralds forth, in no doubtful terms, the principles for which he is resolved to make battle. And battle he does very strenuously make, from the beginning to the end, not only of the present essay, but of his two elaborate and massy volumes. Every "Section," and almost every page of the entire work, is, more or less, an arena of conflict. To vary our style rendering it less warlike, and therefore more appropriate to our subject and purpose, Dr. Paine is a controversial writer; and though we do not, as will appear hereafter, concur with him on every subordinate point he discusses, we consider him, as relates to most, if not all, his fundamental principles and general doctrines, correct and triumphant. The reason of his triumph is plain. He contends for TRUTH, as it is clearly we think expressed in the BOOK OF NATURE, is master of his own subject, and possesses ample means for its illustration and defence. He is, as will be presently perceived, a VITALIST and SOLIDIST. *And so, if we mistake not, is Nature herself.* His course therefore was straight and obvious. And he has pursued it *substantially*, though not, we regret to think, as will be made appear hereafter, in the most direct, conclusive, and desirable manner. He has taken Nature however for his guide, marked her footsteps, followed in her path, and faithfully employed, in vindication of sound principles in medicine, the all-sufficient means, with which she supplies her industrious votaries and enlightened advocates. And thus pursuing his purpose, with ability and perseverance (and he is destitute of neither) his failure to accomplish it may be pronounced *impossible.* For the ultimate triumph of truth is as certain as any of the other decrees of Heaven.

We have said that, in the discussion of his subjects, Dr. Paine pursued the path of Nature. And so he did. But not *directly;* nor with a steady and well-regulated determination to reach his object with the greatest certainty, and the least consumption of time. His course is often exceedingly devious, and his progress therefore *unnecessarily,* not to say, *unreasonably* slow. Instead of selecting the means requisite to *insure* his success (encumbering himself with nothing more) and then advancing with resolution and despatch to the accomplishment of his enterprise; his determination seemed to be, to leave nothing behind him unexplored, that bore the slightest relation to the object he had in view; however useless it might be toward its attainment. Hence resulted a twofold evil. A superabundance of time was wasted by him, in making his *collections* (for they were *selections* no longer;) and, when completed, they were so weighty, that they still farther clogged and retarded his progress, and so cumbrous that he was unable to wield them with the vigor he might have otherwise displayed, and the effect he might have produced. They obstructed the play of his mind, as a plethora of blood does the play of the heart.

In plain terms, our author seems to think that the position he is advocating, whether small or great, complex or simple, intricate or easy, is never sufficiently illustrated and established, until he has said about it every thing that can be said, and quoted, as far as his remembrance extends, (and his memory on the subject of book-knowledge is astonishing) every author that has written on it, either in an ancient or a modern tongue, from the days of Hippocrates to the present period. Nor does he in his citations and references observe, in all cases, the most lucid order. The unfortunate consequence of this mode of discussion is, that he accumulates such a mass of matter, (not a little of it we *think* entirely superfluous) as can hardly fail, as already mentioned, to burden and embarrass his own mind, and detract from the vigor and efficiency of its action. And we *know* that it so bewilders and puzzles, at times, the mind of the reader, as to render him doubtful of the genuine result of the disquisition. When toiling through one of our author's *unnecessarily* learned and protracted arguments, we have felt as if threading a wilderness of trees and under-bush, whose stems and trunks obstructed, while their superabundant foliage darkened our path. Had we not felt confident of the value of what we were in pursuit of (we mean the product of our author's labor) we should have certainly declined the fatigue of searching for it. But the promised worth of the prize cheered us on in the chase; and it is but justice in us to acknowledge, that when the capture was made we were not often disappointed in our anticipation of its value.

In a word; too much light mars the pleasure and distinctness of vision, as certainly as too little. And the same is true of the testimony of quotation. A superabundance of it confounds and darkens. Nor have we ever perhaps witnessed a more decisive confirmation of this truth, in a scientific production in most other respects so valuable, than in that which we are examining. Clearness and condensation are two of the most important qualities of composition and discussion. And they are rarely if ever the issue of extensive reading and clouds of authority. Assuredly they are not the *necessary* issue of them. No; there are perhaps but two methods which argumentative writers and speakers can safely practise. Let them briefly and lucidly state the positions they design to maintain, and adduce in support of them a moderate number of well established facts from nature, and sound authorities from accredited writers—and there stop—or at least be concise, direct, and simple in their inferences. Enlightened and unprejudiced readers and hearers will rarely fail to deduce for themselves the proper conclusions. Or, controvertists and debaters may first advance their facts and arguments, with brevity and perspicuity, in the form of premises, and infer from them as consequences, the principles and doctrines they mean to establish. But let them never over-burden the memories, and fatigue the minds of those whom they wish to instruct or convince, by a superabundance of matter, whether it be derived from nature, or from books. Neither of these methods, however, does our author pursue. While he amasses much more matter than is either necessary or useful, (most of it perhaps sufficiently pertinent,) he observes, in the arrangement of it, nothing of that well-conceived and lucid order, which is so important in the discussion of controverted points—without which indeed obscurity and confusion, rather than light and order, are the perplexing product. But we must close these preliminary remarks, and proceed to the accomplishment of the task we have undertaken.

This however we cannot do, at the present time, in the order, and with the despatch, on which we had calculated. Causes, over which we have no control, compels us to suspend our labours for an indefinite period. We shall close this fragment of an article, with a brief notice of our author's last "essay"—his remarks on the "principal writings of Dr. Louis." That he does not join heartily in the chorus of hosannas, pealed so extensively to the Parisian pathologist, appears sufficiently from the following quotation.

"Our author (Louis) has made all the great lesions of "the typhoid affection," all the constitutional disturbances, and even the cerebral affection, to revolve about the lesion of the glands of Peyer. This is the point of departure, here all the reasoning terminates, and sits enthroned the whole essential pathology of the disease, and for no *other* reason than that the glands of Peyer were found *rather* more frequently altered in structure, than any other part. (p. 682, *Sprengell.*) Little, very little, is allowed even to the disorganized state of the alimentary mucous membrane, or of the liver, or even of the brain itself; and nothing whatever to functional disease, though attended by an injection of the arteries and veins, unless there exist manifest lesions of structure. Indeed it is with our author one constant reiteration of "proportions and disproportions between *the symptoms and lesions of structure*—always terminating in the glands of Peyer." * * * "Whatever display of symptoms our author may have made, his reasoning, his pathology, and even his therapeutics, are founded upon the debris of the body—we mean upon the glands of Peyer. Our author therefore must stand the ordeal by which he has tried and condemned the whole of his profession." * * * "As to the glands of Peyer, the consideration alone which our author has bestowed upon them is a sufficient index to all his medical opinions, and explains abundantly the principles in which the "numerical method" originated, and the therapeutical treatment which he carried out to the gravest inflamations." (Vol. II, pp. 752–3.)

This is but one, out of scores of quotations we could easily make from the Commentaries of Dr. Paine, alike unfriendly to the doctrines of Louis. Indeed the main purport of his essay on the subject, is to discredit, not to say *proscribe* those doctrines, which he assails under the condemnatory title of "*false philosophy.*"

Without gravely attempting, at present, to arbitrate between two writers, one of them so able and learned as the American, and the other so popular and fashionable as the

Frenchman, we say unhesitatingly, that we have long witnessed, with dissatisfaction and regret, the dense and unmeasured clouds of professional incense, that have risen to the latter, from numerous altars in the United States. Such servile *man-worship* (for the practice alluded to amounts to that) if it be not humiliating to the medical character of our country, is sufficiently so to the spirit of those, who so obsequiously minister in it.

What, we ask, has M. Louis done, for the real advancement of practical medicine, that entitles him to such homage? and the only reply which truth countenances, is, NOTHING!! In point of example, he is proverbially one of the *most unsuccessful* practitioners in the metropolis of France. And that is a high standard of comparison. For, in that metropolis, practical medicine, if reports and statistics on the subject be not deceptive, is in a miserable condition—a condition lower than in perhaps any other large European city—and greatly below its standard condition in the towns and villages of the United States.

M. Louis, though professing to be an unprejudiced observer and interrogator of nature, and proclaimed as such, by his pupils and followers, is one of the most confirmed theorists, and exclusive dogmatists, that has appeared in medicine. In his condemnation of the opinions and proceedings of his predecessors and contemporaries, and in assuming that his own method of cultivating medicine *approaches* perfection, if it does not reach it, he is scarcely less sweeping and self-sufficient than Paracelsus. In *his* estimation all, or most, at least, that is professionally valuable, centres in himself, and is incorporated in his "*numerical method*," and his *pathological discoveries.* According to his own estimate of matters, and that of his worshippers, he is the great medical Juggernaut of modern times—or of *all times*—for Æsculapius himself was not we belive a "numerical" physician.

Whatever value may belong to Louis' discoveries in morbid anatomy (and we are not inclined to depreciate them) his scheme of counting is, we feel confident, *immeasurably over-rated.* Never will it—never *can* it issue in the high advantages, in the *treatment and cure of diseases* (the only legitimate end of medicine) which its enthusiastic advocates and admirers expect from it. This we fearlessly proclaim, without pretending to a spirit of prophecy. And future time will confirm the prediction. Nor is this all we have to say on the subject.

Be its fate and fortune what they may, the "numerical method" belongs to M. Louis, not *of right*, but only *by ascription.* It is but *a new name* for an old and valuable attainment in medicine, heretofore denominated *professional experience.* What is experience but the *predominance of observation* as to certain given results? we mean the predominance of the occurrence and existence of certain facts or phenomena, more frequently and generally, than other varying or different phenomena? Most assuredly it is nothing else. And the *amount* of predominance, Louis indicates by *numbers.*

All experienced physicians will say, and have always said, that they have discovered, in certain diseases, certain symptoms to prevail, and certain results to follow the employment of given remedies, and the steady pursuit of given modes of practice. And, adopting a little more precision, on the subject, M. Louis says, I have found these phenomena and results to prevail over others, in the proportion of *fifty* to *twenty;* or in some other proportion equally definite.

Nor do we deny all merit to this increase of "precision,"

by the distinguished Parisian. On the contrary, we acknowledge that it *has* merit; because it tends to the production of greater *accuracy* of observation.

But can M. Louis, by his scheme of figuring, convert the experience of one physician into the experience of another? Can he transfer the experience of the *old* and *practised* physician to the *young* and *unpractised* one? No; he cannot. As well may he attempt, by figures, to transfer the horsemanship and swordsmanship of a Murat to a clown or waterman, who never backed a horse, or drew a sabre. In medicine, as in all things else, man must derive his experience from practice and observation. The recorded experience of one may aid another in acquiring experience; but it cannot *give* him experience, as a *transferable possession.* Though M. Louis may bestow on the profession millions of counts of *his own*, physicians must still continue to observe and count for themselves, else they will never become eminent. Nor is this all. In the treatment of disease, a physician must found his practice not on what he has learnt from others; but on what he perceives to be the condition of patient when he prescribes. As far as he deviates from this rule, he is a mere imitator, and an empiric.

We have said that M. Louis' "numerical method" tends to the increase of accuracy in observation. And so it does. But does not Homœopathy tends to the same effect? Unquestionably it does; and that to an extent beyond perhaps even the "numerical method." One of the strongest features of the homœopathic *system* (if system it may be called) consists in the minuteness and accuracy of observation, which it practises and enjoins. Between the homœpathists and Louis there exists, in one respect, a wide difference. The former direct attention more especially to the *effects of remedies;* the latter to the *symptoms* and *lesions produced by disease.*

In detailing the effects of phosphorus on the human body, one of the homœopathic writers fiills up *twenty-four* large and closely printed octavo pages. By other writers, under the same system, we were told that Magnesia artificialis produces three hundred and twelve symptoms or modes of action—pulsatilla nine hundred and forty—rhus radicans six hundred and fifty—ignatia amara five hundred—arsenic four hundred and sixty—and that certain other articles are not much less prolific in their effects.

In observing and counting, then, we say, we are yet to be convinced that the school of Hahnemann has not an ascendency over the school of Louis. And though we do not mean to insinuate that the two schools are of the same standing in science and merit, we consider them both extravagant and visionary, in some of their notions, and strongly suspect that each wastes time, in pursuit of those notions, which might be better employed on other subjects.

We shall only add, under the present notice, an expression of our hope, that we shall find leisure hereafter to give such farther analyses and expositions of Dr. Paine's "Commentaries," as may be more gratiying and useful to the readers and patrons of this Journal, and more worthy of a work, so erudite and able, as that we have been considering.

